# Eculizumab for acute relapse of neuromyelitis optica spectrum disorder: Case report

**DOI:** 10.3389/fneur.2022.951423

**Published:** 2022-08-08

**Authors:** Sophie Chatterton, John Douglas Edward Parratt, Karl Ng

**Affiliations:** ^1^Department of Neurology, Royal North Shore Hospital, Sydney, NSW, Australia; ^2^School of Medicine, University of Sydney, Sydney, NSW, Australia

**Keywords:** neuromyelitis optica (NMO), demyelination, humanized antibody, case report, neuroimmunology

## Abstract

**Introduction:**

Eculizumab has been shown to be an effective and typically well-tolerated medication in the treatment of neuromyelitis optica spectrum disorder (NMOSD) in maintaining disease remission in patients who are aquaporin-4 water channel autoantibody (AQP4-IgG) seropositive. The efficacy of eculizumab in an acute relapse of NMOSD however is still under review.

**Case:**

We describe a 46 year-old female who presented with acute left monocular vision loss on a background of bilateral optic neuritis treated 15 years prior as suspected NMOSD. She had very poor vision from the right eye (6/60). On presentation she was not on any long-term immunosuppressive agents. Her serum was positive for AQP4-IgG and MRI brain and spine demonstrated areas of demyelination in the corpus callosum and thoracic spine. She was treated with high dose intravenous methylprednisolone and underwent plasmapheresis for five consecutive days, but continued to clinically deteriorate with ongoing blindness in her left eye (light perception only). She was subsequently administered eculizumab with weaning oral corticosteroids. Clinically her vision improved to counting fingers and she remains on maintenance eculizumab infusions in the community. At 3 months, there is a steady improvement but still significant loss of central vision from that eye.

**Conclusion:**

The utility of eculizumab in NMOSD may assist with treating acute episodes. This theoretically accords with the mode of action in inhibiting conversion of C5–C5a/b, perhaps arresting the acute inflammatory process in this disease. Given that disease burden and mortality in NMOSD is almost entirely related to relapses, increased use of eculizumab acutely could potentially aid recovery from an attack in very severe attacks, and therefore minimize immediate stepwise accrual of disability.

## Introduction

Eculizumab is an effective medication approved for the treatment of NMOSD in maintaining disease remission. The efficacy of eculizumab in an acute relapse of NMOSD however remains unknown. We describe a female with NMOSD presenting with acute optic neuritis who continued to deteriorate despite plasmapheresis and intravenous methylprednisolone. She was subsequently treated with eculizumab resulting in a steady improvement in her visual acuity. The clinical utility of eculizumab in NMOSD may extend further than reducing relapse risk and may assist with treating acute episodes. To our knowledge, there is no literature on its use in this setting, only in disease prevention. Given that disease burden and mortality in NMOSD is largely secondary to acute events, increased use of eculizumab earlier in the disease course for acute relapses could potentially minimize morbidity.

## Case presentation

A 46 year-old Chinese female presented to a tertiary Emergency Department with acute left monocular vision loss over the preceding 24 h, associated with painful ocular movements and a severe headache. Her background was significant for suspected NMOSD with initial presentation 15 years previously with bilateral optic neuritis treated with 5 days of pulse intravenous methylprednisolone. At the time, serum AQP4-IgG antibody tests were positive but details lacking. She had migrated overseas and described a history of events concerning for area postrema syndrome with recurrent attacks of intractable nausea and hiccups that had spontaneously resolved. She also described recurrent optic neuritis, with her last episode 18 months previously whilst overseas that was not treated acutely and resulted in permanent significant visual impairment in her right eye. Despite these recurrent events, she was not on any long-term immunosuppression.

Physical examination revealed poor visual acuity in both eyes (6/60 right eye, light perception only left eye). There was a left relative afferent pupillary defect and fundoscopy was significant for right optic nerve head pallor. The remainder of her neurological examination was unremarkable and notably there were no signs of a myelopathic syndrome.

**Figure 1:**
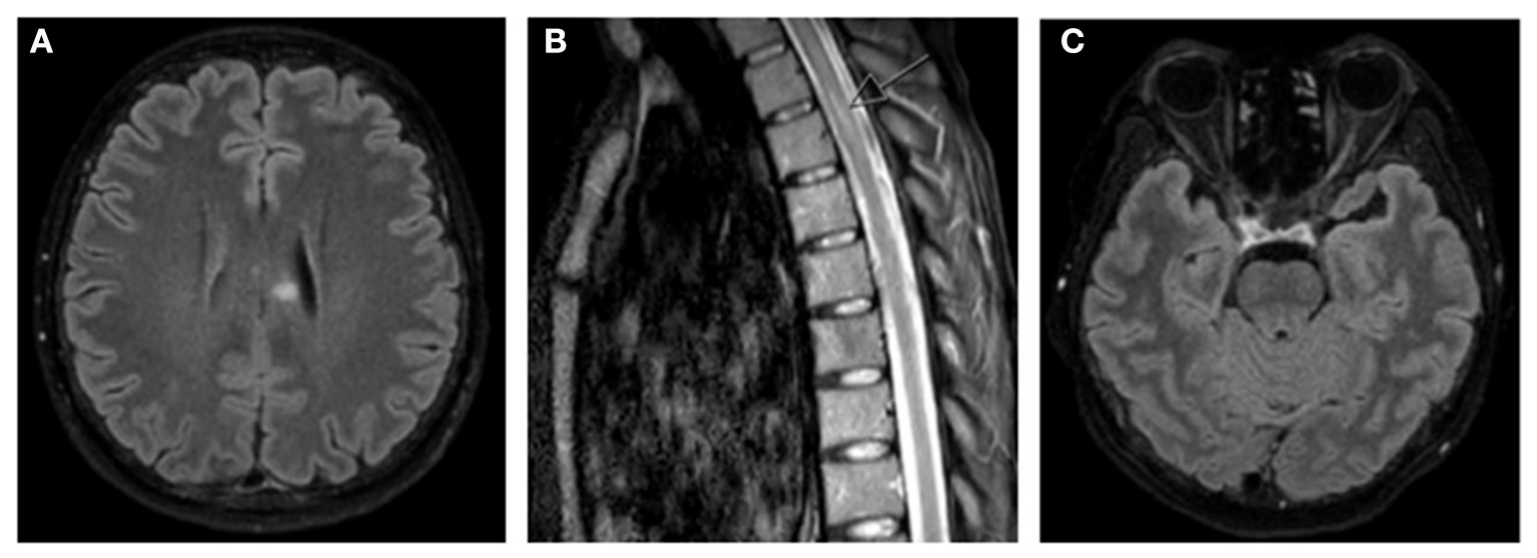
MRI brain and whole spine T2/FLAIR images demonstrating **(A)** hyperintense T2/FLAIR signal in left corpus callosum, **(B)** hyperintense foci in left side of thoracic spinal cord (arrow), and **(C)** hyperintense signal seen in left optic nerve with chiasmal extension with significant atrophy of right optic nerve.

MRI brain and whole spine with gadolinium demonstrated a hyperintense T2/FLAIR signal with restricted diffusion in the left corpus callosum, as well as a hyperintense focus at the level of T5 that was also favored to represent demyelination, neither of which had evidence of active enhancement (Figure 1). High signal with enhancement was seen in the left optic nerve with chiasmal extension and the right optic nerve appeared to have significant atrophy (Figure 1). Repeat serum AQP4-IgG was positive at a greater than screening titer of 1:10 with cell-based indirect immunofluorescence assay (Euroimmun), and the remainder of her blood tests were unremarkable. A lumbar puncture was not performed as the patient declined.

**Figure 2:**
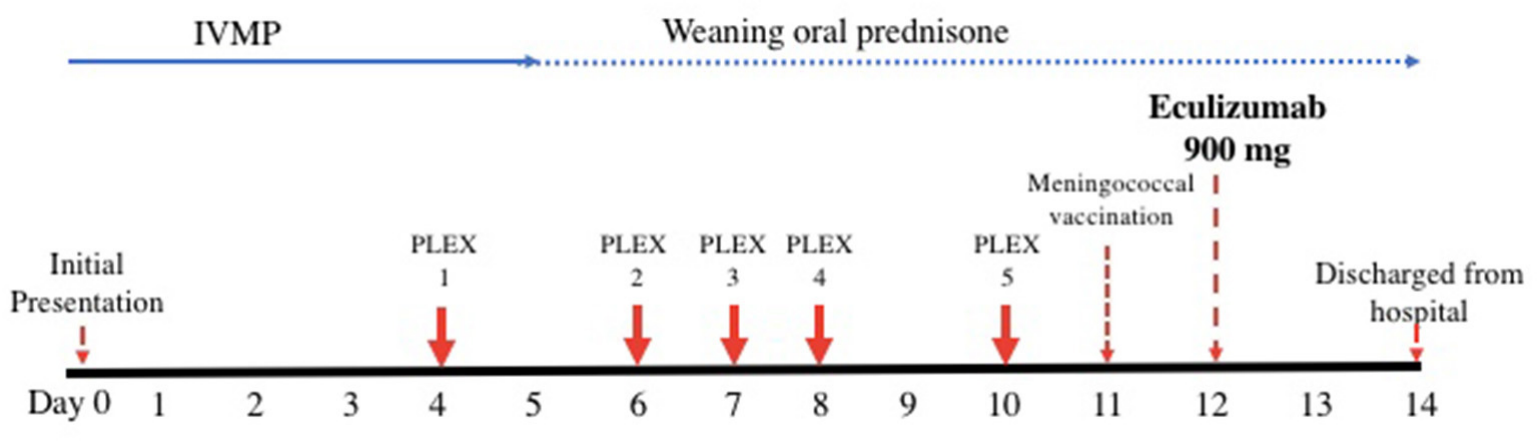
Timeline of acute therapy administered during hospital admission. IVMP, intravenous methylprednisolone; PLEX, plasmapheresis.

Given the typical clinical history and presentation, positive serum AQP4-IgG and neuroradiological findings, the patient was diagnosed with a likely acute relapse of her NMOSD and commenced on pulse intravenous methylprednisolone (IVMP) 1 g once daily for 5 days (Figure 2). Differential diagnoses of her optic neuritis initially considered on presentation included MOG antibody disease, infective etiologies such as bartonella, or other inflammatory conditions such as neurosarcoidosis. These diagnoses were deemed unlikely when her serum AQP4-IgG returned positive.

On her fourth day of IVMP, no improvement in her visual acuity was evident and plasmapheresis was commenced given she was now essentially blind in both eyes with significant disability. She continued to have no improvement in her visual acuity despite two cycles of plasmapheresis and completion of 5 days of IVMP. The main diagnostic challenge at this stage was whether the patient was having a delayed response to her current therapy warranting continuation of her current treatment approach and close observation vs. escalation of therapy.

On day twelve after presentation, she was subsequently administered intravenous eculizumab 900 mg weekly for 4 weeks, then 1,200 mg a week later and fortnightly thereafter. She was also commenced on a slow weaning regimen of prednisolone, starting at 30 mg once daily, decreasing by 2.5 mg every week. On the day prior to administration of eculizumab, she was administered meningococcal vaccinations (MenACWY and MenB) in addition to streptococcal pneumoniae and haemophilus influenza vaccinations as she had not received these before. Amoxicillin 250 mg daily was commenced immediately as meningococcal chemoprophylaxis.

The patient's visual acuity promptly improved to counting fingers with her left eye 2 days after eculizumab commencement and she was able to be discharged home after her first infusion, 2 weeks following her initial presentation. At follow-up 2 and 5 months later, she remained on maintenance eculizumab without complications and there was evidence of ongoing improvement in her left eye visual acuity but still significant loss of central vision. Given the chronicity of her right eye visual impairment, no improvement was expected or found.

## Discussion

NMOSD is a severe autoimmune demyelinating disorder of the central nervous system with a prevalence of 0.3–4.4 per 100,000 population (1). The characteristic features of NMOSD are recurrent episodes of optic neuritis and transverse myelitis, and such attacks typically result in significant disability if not treated promptly (2). Due to the natural progression of NMOSD being that of stepwise deterioration with recurrent attacks and accrued morbidity, long-term immunosuppressive therapy is indicated for the prevention of relapses as soon as the diagnosis is made (3). At present, the mainstay of maintenance therapy for NMOSD is with immunosuppressive therapies, with rituximab traditionally having collectively the greatest efficacy in relapse prevention (4–6). Of note however, there have been no randomized controlled trials comparing the clinical outcomes of different maintenance therapies for NMOSD to date.

Approximately 65–88 percent of patients with NMOSD have positive serum AQP4-IgG antibodies, and it is thought that these antibodies may have a direct pathological role by triggering complement-dependent cytotoxicity (7). Eculizumab is a long-acting humanized monoclonal antibody that binds to terminal complement protein C5 thereby inhibiting breakdown into C5a (which is pro-inflammatory) and C5b (which forms the membrane attack complex) (7). Eculizumab was the first monoclonal antibody to be approved for the treatment of NMOSD. The efficacy of eculizumab was demonstrated in the 2019 PREVENT randomized control trial (RCT) of 143 patients who were AQP4-IgG seropositive with NMOSD that found a significantly lower risk of relapse with eculizumab compared to placebo, with 96.4% of patients in the eculizumab arm relapse free vs. 51.9% in the control arm at 96 weeks (hazard ratio 0.06; 95% confidence interval 0.02–0.20, *p* < 0.001) (7). In comparison, in the RIN-1 RCT of 38 patients who were AQP4-IgG seropositive with NMOSD, no relapses occurred in the rituximab arm vs. seven relapses in the control (placebo) arm at 72 weeks (group difference 36.8%, *p* = 0.0058) (8). Whilst its efficacy has been recently demonstrated, cost remains a significant inhibitory factor for the healthcare system (9) and its efficacy as an acute therapy in NMOSD remains unknown.

Very few studies have compared conventional intravenous methylprednisolone monotherapy with methylprednisolone in addition to plasmapheresis. The response rate to plasmapheresis for NMOSD when high dose methylprednisolone has failed is estimated to be 50–89% (10). Given that the pathophysiology of NMOSD is largely due to a strong humoral response, plasmapheresis is typically deemed the most appropriate therapy in severe relapses (11). Risk factors for poor outcome include delayed presentation or delayed onset of treatment, likely due to these patients sustaining severe, irreversible axonal injury (12).

It is known that up to fifty percent of patients treated with plasmapheresis and/or high-dose intravenous methylprednisolone have a delayed response in improvement in function (10, 13, 14). Therefore, it is possible that the observed effects in our case study may have been at least partially attributable to delayed effect of this therapy, independent of eculizumab use. More studies are needed to explore this further. The use of eculizumab acutely theoretically accords with the immediate mode of action in inhibiting conversion of C5–C5a/b, perhaps arresting the acute inflammatory process in this disease. Given that disease burden and mortality in NMOSD is almost entirely related to relapses, the use of eculizumab acutely could potentially aid recovery from an attack and therefore, minimize accrual of disability. However, this needs to be balanced against the present high cost of this drug, and the difficulty in deciding in the acute phase if there will be a delayed response to traditional first-line acute therapies. Consideration should be given in individual cases to the severity and potential consequence of a given attack, like in this patient, and if continuation of this expensive therapy as prophylaxis is warranted. The place of eculizumab in the treatment of acute attacks however, will require more detailed study, perhaps in a randomized controlled trial setting.

## Data availability statement

The original contributions presented in the study are included in the article/supplementary material, further inquiries can be directed to the corresponding author/s.

## Ethics statement

Ethical review and approval was not required for the study on human participants in accordance with the local legislation and institutional requirements. The patients/participants provided their written informed consent to participate in this study. Written informed consent was obtained from the individual(s) for the publication of any potentially identifiable images or data included in this article.

## Author contributions

SC, JP, and KN: conceptualization and writing—review and editing. JP and KN: supervision. SC: roles/writing—original draft. All authors contributed to the article and approved the submitted version.

## Conflict of interest

The authors declare that the research was conducted in the absence of any commercial or financial relationships that could be construed as a potential conflict of interest.

## Publisher's note

All claims expressed in this article are solely those of the authors and do not necessarily represent those of their affiliated organizations, or those of the publisher, the editors and the reviewers. Any product that may be evaluated in this article, or claim that may be made by its manufacturer, is not guaranteed or endorsed by the publisher.
